# Smart emulsion system driven by light‐triggered ionic liquid molecules and its application in eco‐friendly water‐saving dyeing

**DOI:** 10.1002/smo.20230030

**Published:** 2024-03-19

**Authors:** Aiqin Gao, Jiahui Liang, Mingxiao Jing, Xiyu Song, Aiqin Hou, Kongliang Xie

**Affiliations:** ^1^ State Key Laboratory for Modification of Chemical Fibers and Polymer Materials College of Chemistry and Chemical Engineering Donghua University Shanghai China; ^2^ College of Textile and Apparel Shaoxing University Shaoxing China

**Keywords:** ionic liquid, liquid crystals, molecule switch, photo‐response, smart emulsion

## Abstract

The smart emulsification and demulsification system with the light response is a useful tool in various industries, including green chemistry, catalytic reaction, pharmaceuticals, and environmental remediation. Herein, an ionic liquid crystal compound with a light triggered switch based on the azobenzene group [(4‐{3‐methyl‐1‐[3‐(8‐octyloxyoctyl)oxy‐4‐oxobutanoyl]imidazo‐lium‐1‐yl}octyl)oxy] ‐N‐(4‐methylphenyl)benzene‐1,2‐diazene bromide (MOIAzo), was designed and synthesized, which could cause reversible transition between emulsification and demulsification through the light trigger. The ionic liquid has an efficient photoinduced liquefaction process, which dramatically lowers the melting point of ionic liquids from 79 to 9.2 ^o^C. This significantly broadens the liquid state temperature of the ionic liquid crystal. The ionic liquid crystal MOIAzo exhibits both photoinduced and thermally induced nematic liquid crystal properties. The smart emulsion system was effectively employed in an eco‐friendly water‐saving dyeing process of cationic dyes for cationic dyeable polyester (CDP) fabrics, which used only half the amount of water compared with the conventional water bath dyeing method. After dyeing, the oil and water phases can be efficiently separated through the light irradiation, and the oil phase can be reused for the subsequent dyeing process. This novel smart emulsion dyeing method greatly reduces the water consumption and wastewater discharge. MOIAzo as a light‐triggered ionic liquid molecule opens up new dimensions in green chemistry.

## Graphical abstract

An ionic liquid crystal compound with a light‐triggered switch was designed. The smart molecule can efficiently transform between the solid and liquid states at the ambient temperature and can reversely emulsify and demulsify oil and water mixtures through the light‐triggered switch to create a smart emulsion system.

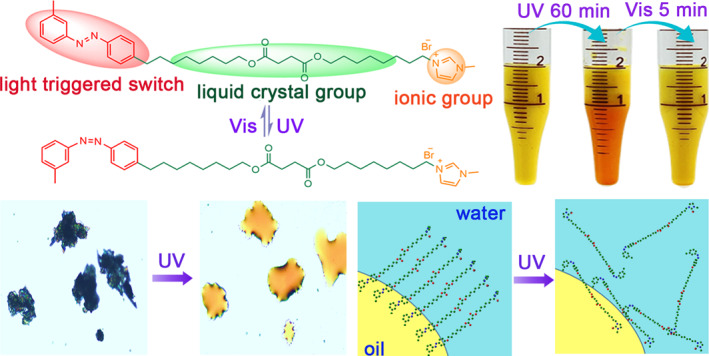

## 1 INTRODUCTION

Ionic liquids (ILs) are a class of salts with unique physicochemical properties, such as low volatility, excellent thermal stability, and remarkable solvation capabilities.[^1^, ^2^, ^3^] These attributes have made ILs attractive for a wide range of applications, including solvent replacements, catalysis, biomass conversion and energy storage.[^4^, ^5^, ^6^] In recent years, various functionalized ionic liquids have gained significant attention.[^7^, ^8^, ^9^, ^10^] The versatile nature of functionalized ionic liquid molecules positions them as an exciting class of materials with the considerable potential for application in diverse fields,[^11^, ^12^, ^13^, ^14^] including the green synthesis of pharmaceuticals, fine chemicals, and polymers.[^15^, ^16^, ^17^] This can lead to the increased reaction rates, selectivity, and yield, thereby minimizing the need for harsh reaction conditions. ILs can act as functional surfactants, lowering the interfacial tension between immiscible phases. This property is particularly useful in separation processes, such as liquid‐liquid extraction or biphasic catalysis, as it enables efficient phase separation and recycling of reactants.[^18^, ^19^, ^20^] ILs have been successfully employed in various separation processes, such as the extraction of biomass, metals and organic compounds, due to their high selectivity and efficiency.[^21^, ^22^, ^23^, ^24^]

Recently, the stimulus‐responsive reversible transition between emulsification and demulsification has become a vibrant field of interest. It harnesses the power of various stimuli to reversibly control the emulsification and demulsification processes, potentially bringing remarkable enhancements in some industries, including food, cosmetics, pharmaceuticals, and environmental remediation.[^25^, ^26^, ^27^, ^28^] Many of these systems are dictated by the presence of smart materials, such as responsive polymers, nanoparticles, and surfactants, which undergo conformational changes in response to external stimuli, such as temperature,^[29]^ magnetic field,^[30]^ pH variation,^[31]^ CO_2_/N_2_,[^32^, ^33^] light irradiation^[34]^ and their combination,^[35]^ thus affording the unprecedented control over the emulsification‐demulsification process. These systems respond to external triggers, enabling the on‐demand control over the emulsion stability. Ionic liquids are promising green solvents for controlling the emulsion stability and separation due to their tunable nature and versatility in structure and functionality. He et al. reported the use of ILs as a dual‐responsive emulsion microreactor for the Knoevenagel condensation reaction.^[36]^ The use of stimuli‐responsive ILs enables the reaction to be performed in the homogeneous phase and facilitates easy separation of products and catalysts in the heterogeneous phase. Wang et al. designed a new type of ILs microemulsion sensitive to temperature changes, allowing for the reversible emulsification and demulsification.^[37]^ The same group conducted a light‐responsive Pickering emulsion stabilized by Pd‐supported silica nanoparticles and azobenzene ionic liquid surfactant. The emulsion could be used as a micro‐reactor for catalytic reactions, with the ability to separate products and recycle the emulsifier and catalyst.[^38^, ^39^, ^40^] However, because the melting point and molecular configuration of these ionic liquids are not easily adjusted, it is difficult for the emulsion system to efficiently and reversibly emulsify and demulsify at room temperature.[^41^, ^42^] Designing novel ionic liquid molecules to construct sensitive and efficient smart emulsion systems remains a challenge.

**FIGURE 1:**
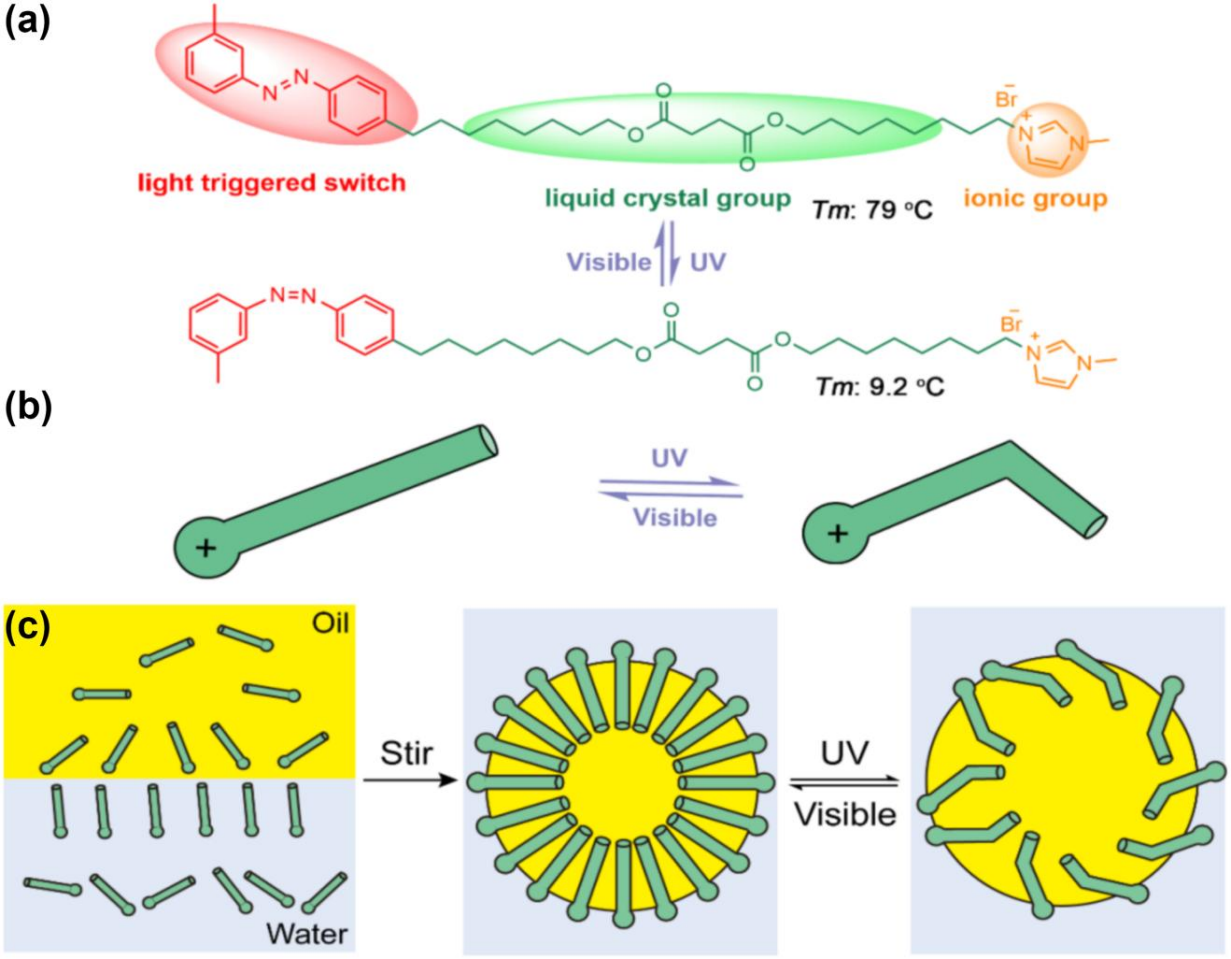
Schematic diagrams of MOIAzo and the designed smart emulsion conversion process during UV and visible light irradiation. (a) Trans‐cis isomerization of MOIAzo. (b) Schematic diagrams of MOIAzo configuration change. (c) Smart emulsion conversion.

Herein, we design a smart emulsion system driven by a light‐triggered ionic liquid molecule that can perform the emulsification and demulsification conversion efficiently at room temperature under the light induction. A light‐triggered ionic liquid crystal molecule containing photo‐responsive azo group, 4‐[(4‐{3‐methyl‐1‐[3‐(8‐octyloxyoctyl)oxy‐4‐oxobutanoyl]imidazolium‐1‐yl}octyl)oxy]‐N‐(4‐methylphenyl)benzene‐1,2‐diazene bromide (MOIAzo), is designed and synthesized, which undergoes trans‐cis isomerization under the UV and visible light. MOIAzo can efficiently transform between the solid and liquid states at room temperature. This is important because, in the liquid state, the molecular configuration of the ionic liquid is more flexible. Such flexibility allows for easier and more efficient adjustment of the molecular structure in response to external stimuli (like light in this case), which is necessary for the reversible transition between emulsifying and demulsifying states. The ionic liquid molecule containing liquid crystal group is easy to form emulsion micelles due to its amphiphlic performance. This structure change gives it the ability to cause photo‐induced reversible transition between the emulsification and demulsification. The schematic diagrams of the designed smart emulsion conversion process using the ionic liquid crystal molecule MOIAzo are shown in Figure 1.

The liquid crystal and solid‐liquid conversion properties of the light ‐ triggered ionic liquid crystals were investigated, and the mechanism of the efficient emulsification and demulsification conversion was also discussed. The smart emulsion was employed in an eco‐friendly water‐saving dyeing process of cationic dyes for cationic dyeable polyester (CDP) fabrics.

## 2 MATERIALS AND METHODS

### 2.1 Materials

All chemicals used in the synthesis of MOIAzo were purchased from Sinopharm Chemical Reagent Co., Ltd., Shanghai, China. They were used without the further purification. The cationic dyes, Brilliant Red SD‐5 GN, Yellow SD‐5 GL, and Blue SD‐GB, were obtained from Dongwu Chemical Co. Ltd., Suzhou, China. The CDP fabric was purchased from Zhejiang Shengfa Textile Co. Ltd., Huzhou, China.

### 2.2 Synthesis and characterization of MOIAzo

The synthesis procedures and characterization of MOIAzo and the intermediate compounds are available in the Supporting Information (Figure S1–S8).

### 2.3 Preparation of paraffin‐water smart emulsion

A predetermined concentration of MOIAzo was added to a certain volume of paraffin and stirred to disperse evenly, and then an equal volume of deionized water was slowly added. The mixture was then emulsified for 15 min at 8000 rpm using a high‐speed shearing dispersion homogenizer (IKA T 25 digital ULTRA‐TURRAX) to obtain a paraffin‐in‐water (50/50, O/W) smart emulsion. The emulsion was then placed in the dark and left undisturbed for over 8 h to observe the stability. The preparation process for toluene‐in‐water and heptane‐in‐water emulsions was similar to that of the paraffin‐in‐water emulsion, except that paraffin was replaced with an equal volume of toluene or heptane.

### 2.4 Photo‐responsive emulsification and demulsification of the paraffin‐water emulsion

A stable paraffin‐water emulsion (2 mL) was placed in a graduated glass tube and irradiated with a 365 nm UV lamp (innuoK10, 20 W/m^2^) to observe the demulsification of the smart emulsion. The demulsification efficiency was calculated based on the volume of the separated water phase. The emulsion was then irradiated with a daylight lamp (SHENYU 1007‐50W, 100 W/m^2^) and the stability of the emulsion was observed after the homogenization.

### 2.5 Emulsion dyeing of cationic dyes on cationic dyeable polyester fabric

The stable paraffin‐water emulsion (50/50) was prepared with the concentration of the MOIAzo compound at 0.5 g/L as described above method. Cationic dyes, Brilliant Red SD‐5 GN, Yellow SD‐5 GL, and Blue SD‐GB, were selected to dye CDP fabric using the smart emulsion. The dye concentration was 2% (o.w.f) based on the weight of the fabric. The dyeing bath ratio was 1:10, dyeing started at 30 ^o^C, and the temperature was raised to 125 ^o^C at a rate of 2 ^o^C/min, followed by dyeing at this temperature for 50 min. Afterwards, the dyeing residue was cooled to the room temperature, the fabric was removed and washed with water, then soaped at 85 ^o^C in a 1 g/L soap solution for 15 min. After being removed, it was washed with cold water and dried. To compare emulsion dyeing with the conventional water bath dyeing, the fabric was also dyed in a water bath under the same conditions, with the same bath ratio, dye concentration, and dyeing process, with water as the dyeing medium.

### 2.6 DFT calculation

The trans‐ and cis‐configurations of MOIAzo were calculated using the Gaussian 16 software, using the B3LYP hybrid function and the 6–311 G (d, p) basis set.

### 2.7 Tests and characterization

The structures of MOIAzo and the intermediate compounds were characterized using infrared spectroscopy, ^1^H NMR, and ^13^C NMR. Infrared spectroscopy was performed on a Spectrum Two Fourier Transform Infrared Spectrometer (PerkinElmer, USA). ^1^H NMR and ^13^C NMR were carried out on an Avance3hd600mhz NMR spectrometer (Bruker, Germany). UV‐visible spectroscopy was performed using a U‐3310 UV‐visible spectrophotometer (Hitachi, Japan). Optical microscope images were taken using a DM2700 P Leica hot stage polarizing microscope (Leica, Germany). The surface tension was measured using a CNSHP BZY‐2 automatic interface tension meter (Hengping Instrument, China). Particle size was measured using a NANO ZS nanoparticle size potential meter (Malvern, UK).

The dyeing of CDP fabric was performed in an AHIBA IR PRO high‐temperature, high‐pressure dyeing machine (Datacolor, USA), and the K/S values of the dyed fabric were measured using a DATACOLOR 650 color matching device (Datacolor, USA).

## 3 RESULTS AND DISCUSSION

### 3.1 Photoinduced liquefaction and its liquid crystal texture of the ionic liquid crystals

Photo‐responsive compounds have been widely studied due to their intriguing photo‐isomerization features when irradiated with UV/Visible light.[^40^, ^43^, ^44^] By introducing the ionic liquid structure into the liquid crystal molecular structure, a new kind of ionic liquid crystal (ILC) with anisotropic properties can be designed. Here, we design an ionic liquid crystal molecule MOIAzo which contains a photo‐responsive azo group and an esterified liquid crystal group with a long carbon chain. The cationic methylimidazole and azo groups are located at either end of the long carbon chain esterified group. Ionic liquid molecules containing liquid crystal groups is easier to make the molecular configuration tend to be in an oriented arrangement.

**FIGURE 2:**
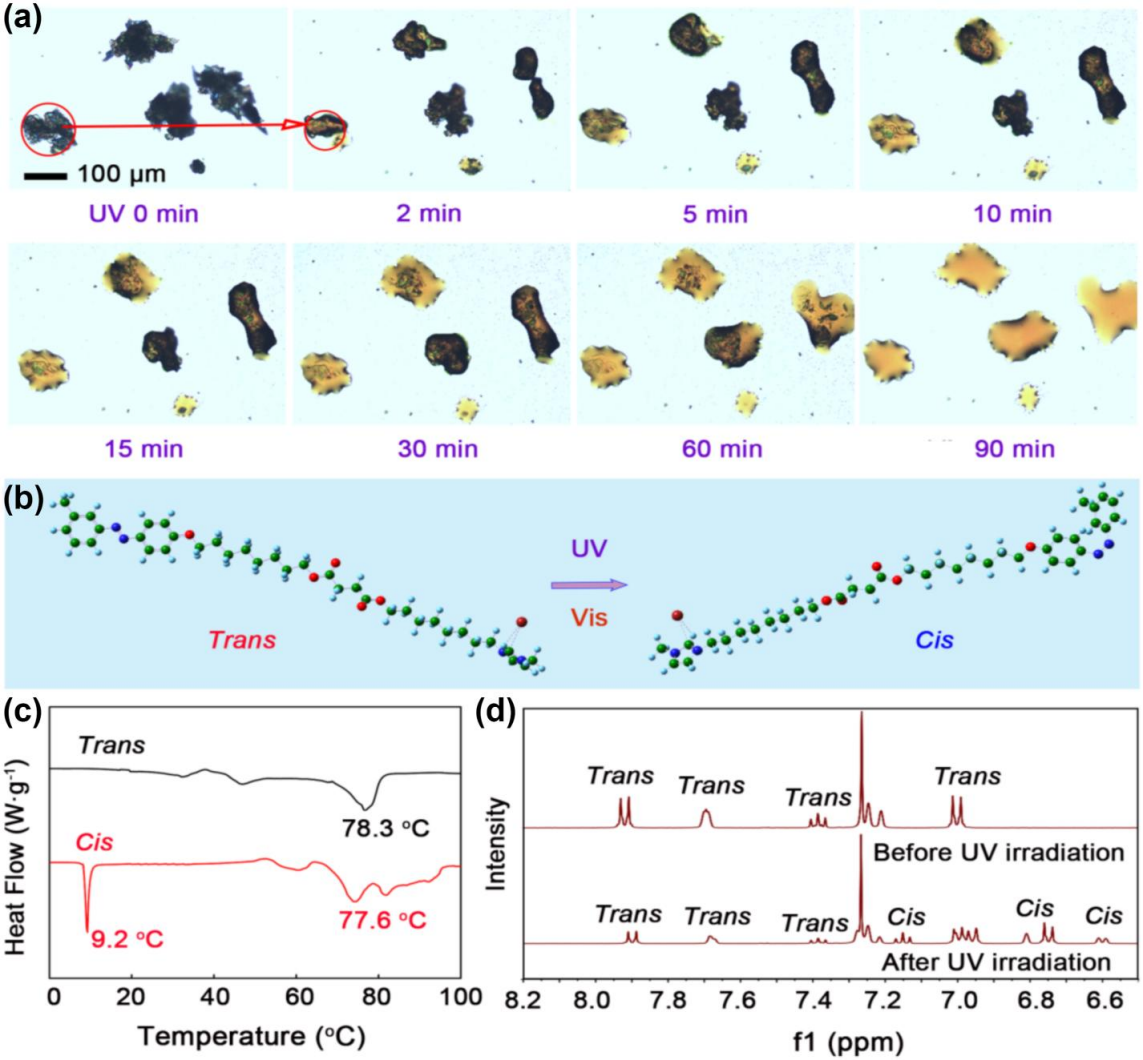
Photoinduced liquefaction of the ionic liquid crystal MOIAzo and its photoisomerization process. (a) OM images of reversible solid‐liquid transitions of MOIAzo under the different time UV irradiation. (b) Configuration isomerization optimized using Gaussian software. (c) DSC of the MOIAzo photoisomerization. (d) ^1^H NMR spectra of MOIAzo before and after the UV irradiation.

The melting point of the synthesized MOIAzo compound was 79–80°C. MOIAzo exhibited a remarkable solid‐to‐liquid transition induced by the UV light irradiation at room temperature. The solid‐to‐liquid transition process was studied by observing the morphology transformation of the solid powder on a glass substrate after the different time UV irradiation, as shown in the optical microscope (OM) images in Figure 2a. After the UV irradiation for 2 min, the thinner regions of the MOIAzo solid powder began to melt, changing from an irregular powder morphology into droplet shapes, indicating the liquefaction of the MOIAzo compound, as shown by the red circles and arrow. As the UV light irradiation time increased, more regions liquefied from solid to liquid. After the irradiation for 90 min, all the solid powder converted into droplets, indicating the complete liquefaction of the ionic liquid crystal MOIAzo. The difference in the photoinduced liquefaction time in different regions is due to the variations of the sample thickness. The results show that the melting point of the ionic liquid MOIAzo can be reduced rapidly under the light induction, and they can be magically transformed from solid state to liquid state in a short time. This significantly broadens the liquid state temperature of the ionic liquid crystal. In order to exclude the effect of temperature changes caused by light, the control experiments were performed to study the photoinduced solid‐to‐liquid transition. Before the UV irradiation, the temperature of MOIAzo was 30.9 ^o^C monitored by an infrared thermometer. After the UV irradiation (20 W/m^2^) for 90 min, the surface temperature raised to 33.5 ^o^C (Figure S9). To compare, MOIAzo powder was heated to 33.5 ^o^C and for 90 min, it was not liquefied, demonstrating that the photoinduced solid‐to‐liquid transition was due to the photoisomerization instead of a photothermal process.

The MOIAzo molecule with the light‐trigger undergoes trans‐cis isomerization under the UV and visible light. The two molecular configurations during the isomerization process are optimized using Gaussian software and shown in Figure 2b. In the trans configuration, the two aromatic groups are on opposite sides of the ‐N=N‐ double bond, leading to a more linear, rod‐like molecular shape, while in the cis configuration, the two aromatic groups are on the same side of the ‐N=N‐ bond, leading to a more bent, V‐shaped molecular geometry. DSC measurements were further performed to illuminate why MOIAzo was liquefied under the UV irradiation at room temperature (Figure 2c). The trans MOIAzo shows a phase transition temperature at 78.3 ^o^C. The cis MOIAzo has a broad exothermic band at ∼77.6 ^o^C due to the thermal cis‐to‐trans isomerization during the heating process, resulting in a mixture of both cis and trans structures, which is why a broad peak appears. The low phase transition temperature of cis MOIAzo below room temperature at around 9.2 ^o^C is in accordance with the liquefied phenomenon that cis MOIAzo was liquefied at room temperature. Thus, it can be concluded that a liquefaction temperature difference (70 ^o^C) between trans‐ and cis‐ MOIAzo contributes to the photoinduced solid‐to‐liquid state transition. Since cis‐azobenzene and trans‐azobenzene have different proton chemical shifts, ^1^H NMR spectra further demonstrate the photoisomerization properties (Figure 2d). After the UV light irradiation, new peaks appear near 6.60, 6.80, and 7.15 ppm, as a result of the photoinduced trans–to–cis transition. However, the trans structure cannot be completely converted to the cis structure. This is because the trans isomer is more stable, and during the testing process, some cis isomers continuously convert back to the trans isomers.

**FIGURE 3:**
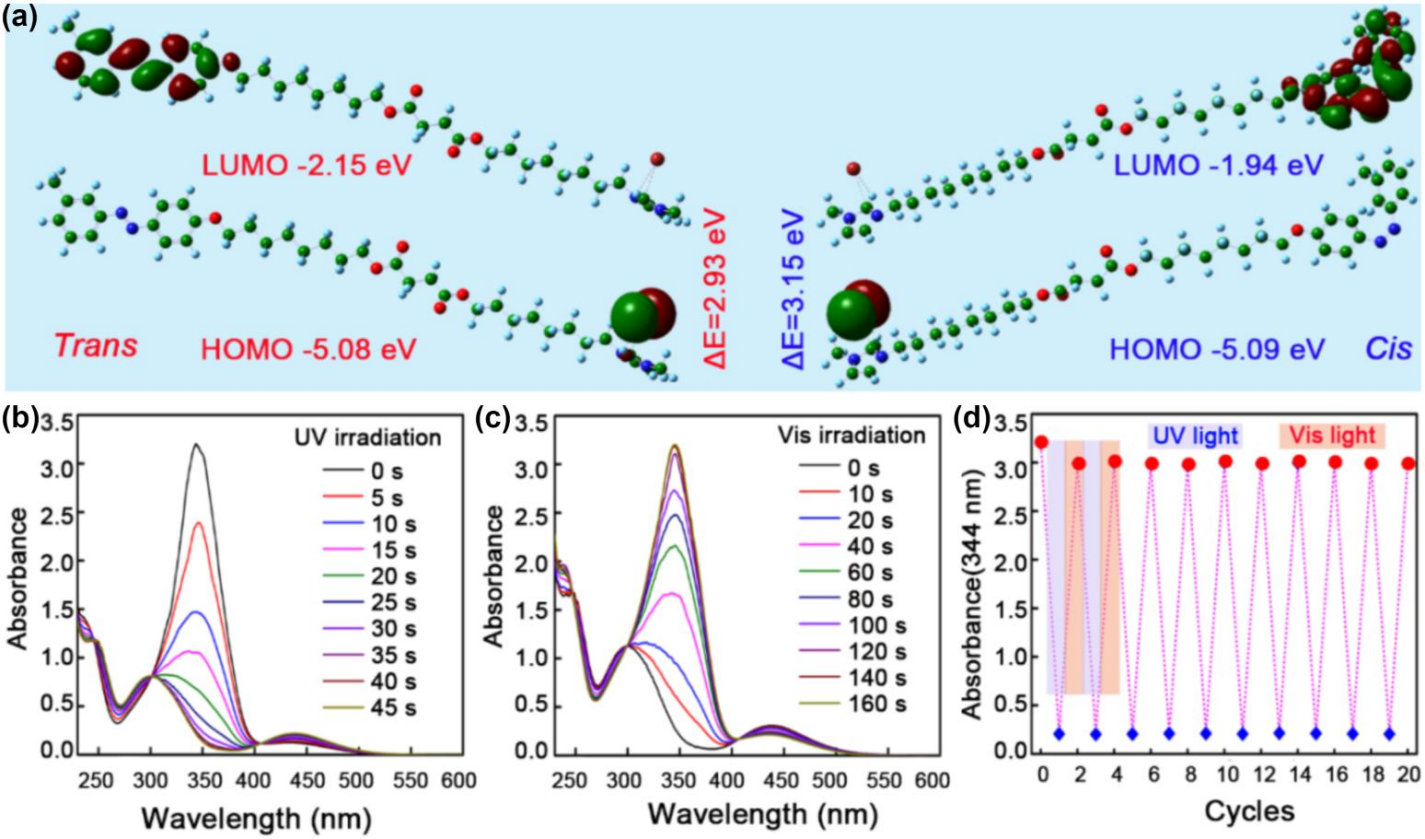
Calculated molecular orbitals of the ionic liquid crystal MOIAzo and its UV‐vis absorption spectra. (a) Calculated molecular orbitals of trans‐ and cis‐ MOIAzo. (b, c) UV‐vis absorption spectra of MOIAzo under 365 nm UV and visible light irradiation, respectively. (d) Evolution of absorbance at 344 nm upon the UV and visible light irradiation.

The trans‐cis isomerization of the ionic liquid crystal molecules MOIAzo can be explained from the perspective of molecular orbital energy levels, so the molecular orbitals containing the main electronic transitions of MOIAzo were calculated based on the optimized ground states and shown in Figure 3a. In the trans configuration, all π‐electrons reside in the π orbital with lower energy. When MOIAzo absorbs the UV light, the energy of the photons is absorbed by the electrons within the molecule, exciting the electrons from the lower energy HOMO orbital (−5.08 eV) to the higher energy LUMO orbital (−2.15 eV). This means that π‐electrons are excited from the π orbital to the π* orbital, altering the distribution of the molecular electron cloud. This alteration in the electron cloud distribution leads to a change in the molecular structure. As electrons excited from the π orbital to the π* orbital, the molecule enters a high‐energy state, which is unstable. Subsequently, the molecule undergoes internal rotation, seeking a new and more stable state, thereby accomplishing the transition from the trans configuration to the cis configuration.

The absorption of UV/visible photons can induce trans‐cis photoisomerization and the process can be tracked by UV‐vis absorption spectra. The UV‐vis absorption spectra of MOIAzo under the UV and visible light irradiation are shown in Figure 3b,c. Both the trans and cis isomers exhibit a π→π* absorption band in the UV region around 344 nm and a less intense n→π* absorption band in the visible region around 433 nm. After the UV light irradiation, the absorption at 344 nm drops and that at 433 nm enhances. The trans‐MOIAzo reaches the photostationary state (PSS) after theUV irradiation for 40 s. Figure 3c shows an inverse trend, which is the reversible isomerization process from cis to trans configuration under the visible light irradiation for 140 s. The absorption intensity at 344 nm is almost the same as that before the UV irradiation, indicating that the isomerization capacity does not decrease after one cycle of the light irradiation. The enduring trans‐cis‐trans isomerization cycle of MOIAzo is analyzed for prolonging the reusable function. The MOIAzo solution undergoes the UV illumination until reaching a cis PSS, and then the visible light irradiation isomerizes it back to a trans‐form PSS (Figure 3d). The isomerization degree of trans or cis configurations remains constant after 20 switch‐cycles. The repeated alternation of PSS demonstrates an impressive fatigue resistance.

**FIGURE 4:**
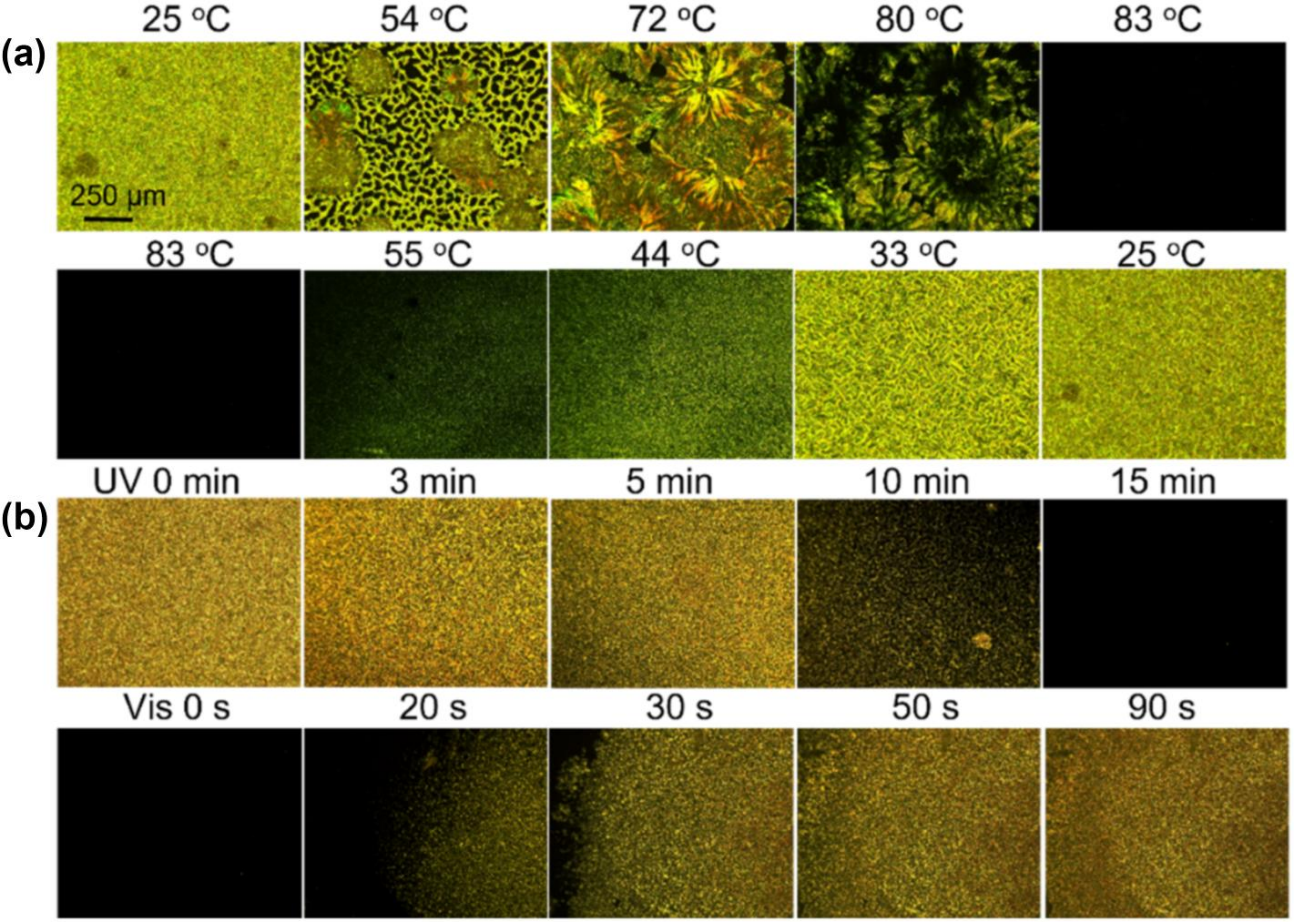
The nematic liquid crystal texture of MOIAzo. (a) Polarizing optical microscope (POM) images during the heating and cooling process. (b) POM images under the UV/Vis irradiation.

In addition to the photoinduced solid‐to‐liquid transition, the MOIAzo molecule also exhibits thermochromic liquid crystal and photoinduced liquid crystal properties. Ionic liquid molecules contain liquid crystal groups, which make the molecules in an oriented arrangement. The MOIAzo solid powder was placed under a polarizing optical microscope (POM) and subjected to heating and cooling at a rate of 1°C/min. As shown in Figure 4a, during the heating and cooling process, we observed a nematic liquid crystal in the temperature range from room temperature to 82°C. This is because the trans‐structure of the molecular chain has an anisotropic elongated conformation. As the temperature rises above the phase transition temperature, the crystalline region transforms into a disordered arrangement, and the molecular chains adopt a random conformation, the liquid crystal molecules begin to transition to an isotropic liquid state. At a temperature of 83°C, along with the transition from the liquid crystal phase to the isotropic state, it appears as the disappearance of the liquid crystal texture under POM. Upon cooling below the phase transition temperature, the crystalline region spontaneously recovers its orientation, and the shape of the liquid crystal compound returns to its initial state again. The liquid crystal appeared a heterogenous crystalline phase during the heating process, which was caused by the reorientation of the molecules. When heated, the energy input can disrupt these arrangements, causing the molecules to reorient themselves. This reorientation alters the optical properties of the material, leading to changes in its texture and appearance. Usually, heating can transition the material from a more ordered phase to a less ordered one.

The trans‐configuration of the azobenzene molecule is a lower energy rod‐like structure and is thermodynamically stable. It is compatible with the ordered liquid crystal phase and easily forms a well‐ordered nematic phase under appropriate alignment conditions (Figure 4b). Under the UV light irradiation, the azobenzene undergoes a cis‐trans isomerization, transition from the trans‐to the cis‐configuration. The cis‐azobenzene molecules exhibit a bent structure, reducing the overall alignment of the liquid crystal system and causing a phase transition from the liquid crystal phase to the isotropic state, resulting in significant macroscopic deformation of the liquid crystal system. Due to the large steric hindrance and instability of the cis‐azobenzene, it easily reverts back to the rod‐like trans‐structure under the visible light irradiation. The liquid crystal state texture of the ionic liquid compound MOIAzo also has a sensitive reversible response to the light stimulation. The ionic liquid crystal compound undergoes both thermal and photo rearrangement to form nematic liquid crystal textures.

### 3.2 Smart emulsification and demulsification oil‐in‐water using the ionic liquid crystal MOIAzo

**FIGURE 5:**
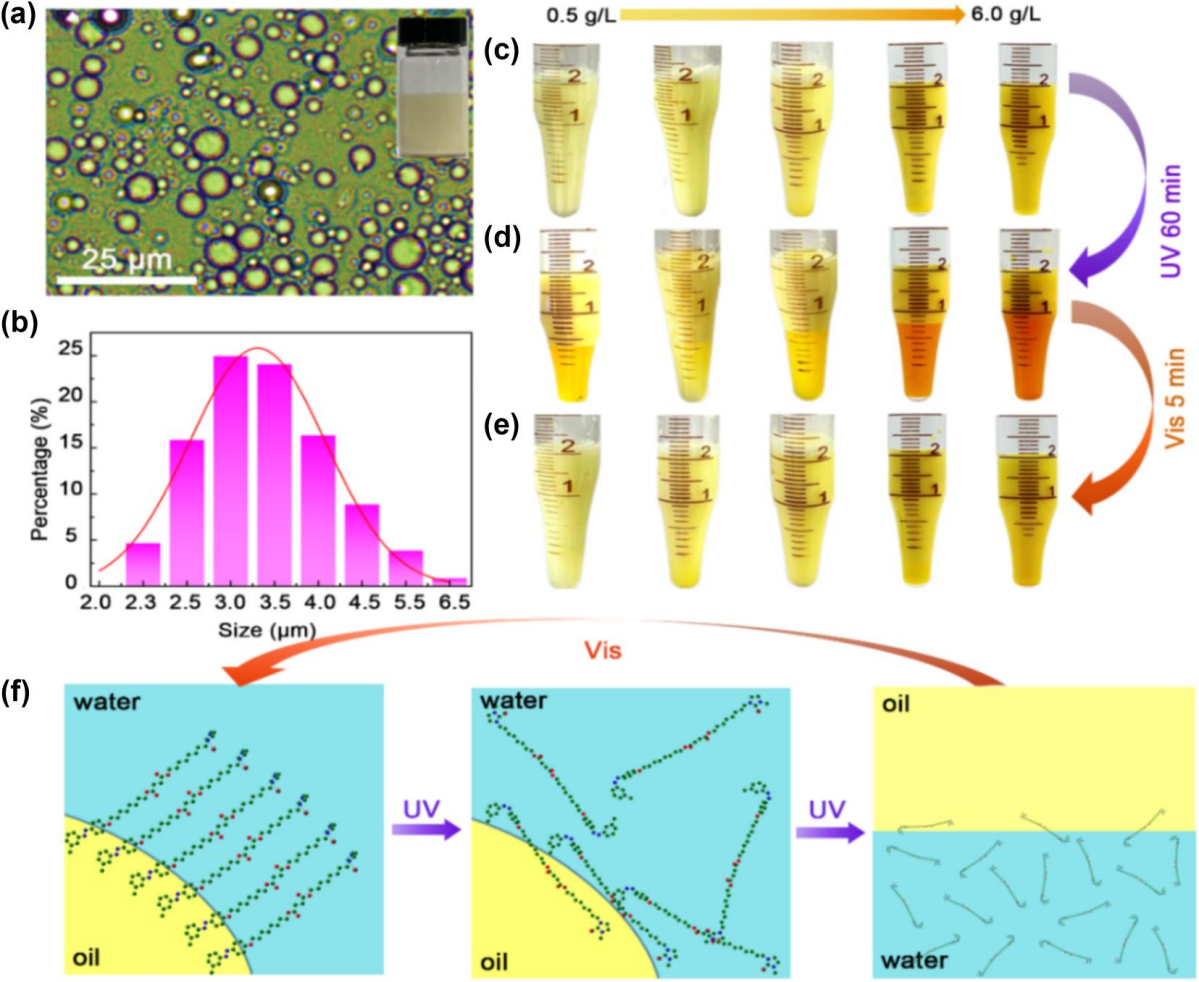
Light‐induced emulsification and demulsification in the MOIAzo System. (a) Microscopic view of a paraffin‐water emulsion stabilized with 0.5 g/L MOIAzo, including a photo of the bulk emulsion. (b) Analysis of particle size in the paraffin‐water emulsion. (c) Photos of the paraffin/water emulsion at different MOIAzo concentrations. (d) Demulsification under 60 min of UV light exposure. (e) Emulsification under 5 min of visible light irradiation. (f) Schematic of the photo‐triggered emulsion process, detailing the demulsification mechanism.

Because the cationic methylimidazole and the esterified liquid crystal group with azo group are located at either end of the molecule, the ionic liquid crystal MOIAzo possesses an amphiphilic performance of the functional surfactant. It has excellent surface activity, which can greatly reduce the surface tension of the solution. MOIAzo could be employed as an emulsifier to fabricate smart emulsions. To determine the emulsifier concentration, the critical micelle concentration (CMC) of MOIAzo was first measured. The change in surface tension of MOIAzo aqueous solution with increasing the concentration is shown in Figure S10, from which the CMC of MOIAzo calculated was 0.19 g/L. We prepared the paraffin‐in‐water emulsion at a MOIAzo concentration of 0.5 g/L, above the CMC. Figure 5a shows the optical microscopic photograph of the paraffin‐in‐water emulsion. The volume ratio of the paraffin and water is 1:1 (50/50). The optical microscope image indicates that the droplets are polydisperse with several micrometer diameters. The particle size distribution chart shows more intuitively that the size of the emulsion droplets is mainly distributed between 2 and 6.5 μm, with the majority of particles having a diameter between 3 and 3.5 μm (Figure 5b). MOIAzo has also been proved to be suitable for preparing toluene‐in‐water and heptane‐in‐water emulsions, and optical microscope images of the emulsions are shown in Figure S11. The particle size distribution chart indicates that the droplet sizes of the toluene‐in‐water emulsion prepared with MOIAzo are mainly distributed between 0.95 and 1.1 μm, while the droplet sizes of the heptane‐in‐water emulsion are mainly distributed between 1.7 and 2.0 μm. The thermal stability of the MOIAzo‐based paraffin‐water emulsion was evaluated at a MOIAzo concentration of 0.5 g/L at room temperature and a high temperature of 60 ^o^C. Results (Figure S12) show that the emulsion can remain stable for 60 min under the high temperature.

Interestingly, the stable emulsion can be demulsified resulting in oil and water phase separation upon the UV irradiation for 60 min at room temperature. Subsequently, a stable emulsion can also be restored by the irradiation with the visible light for 5 min after the homogenization. Figure 5c shows the photographs of paraffin‐in‐water emulsions prepared with different concentrations of MOIAzo. The emulsion volume is 2 mL, comprising 1 mL of water and 1 mL of paraffin. From left to right, the concentrations of MOIAzo are 0.5 g/L, 1.0 g/L, 2.0 g/L, 5.0 g/L, and 6.0 g/L, respectively. We observed that MOIAzo could effectively stabilize the emulsion at a concentration higher than 0.5 g/L. Next, the emulsions were placed under 365 nm UV light with an intensity of 20 W/m^2^ for 60 min. From Figure 5d, it is observed that phase separation occurred between the oil and water phases in the emulsions. Higher MOIAzo concentrations led to more efficient phase separation, and at a concentration of 6.0 g/L, a complete separation of the oil and water phases took place. Subsequently, the emulsions with oil‐water phase separation were exposed to white light and homogenized. It is found that the emulsions quickly returned to a stable state, as shown in Figure 5e. Therefore, we could achieve reversible switching between a stable emulsion and demulsification by the alternate exposure to the UV and visible light. A smart emulsion of oil‐in‐water using the ionic liquid crystal MOIAzo was obtained.

The possible mechanism of the light‐induced emulsification and demulsification is illustrated in Figure 5f. In a stable state, the MOIAzo molecule is a trans conformation, forming a linear structure. At this time, MOIAzo molecules are densely arranged, forming a stable interfacial layer between oil and water. However, when exposed to the UV light, the molecular conformation changes from trans to cis and the molecular structure changes from linear to bend. The azophenyl group connected to the liquid crystal chain segment begins to be folded, and the hydrophobic segment becomes shorter. The molecules can no longer form a dense protective layer at the oil‐water interface. Therefore, the emulsion gradually gets destroyed and more MOIAzo molecules enter the water phase, ultimately leading to the separation of oil and water phases. When further irradiated with the visible light, the molecules change back from cis to trans conformation, and under the effect of homogenization, they can form a dense interface layer to stabilize the emulsion again. The particle size change during the photo‐switching processes has been supplemented using optical microscopic measurements. As the illumination time increases, the size of the emulsion droplets gradually becomes larger, until they aggregate into large droplets of several tens to hundreds of micrometers, resulting in the destruction of the emulsion (Figure S13).

### 3.3 Application of the paraffin/water smart emulsion with light‐triggered switch for eco‐friendly water‐saving dyeing

**FIGURE 6:**
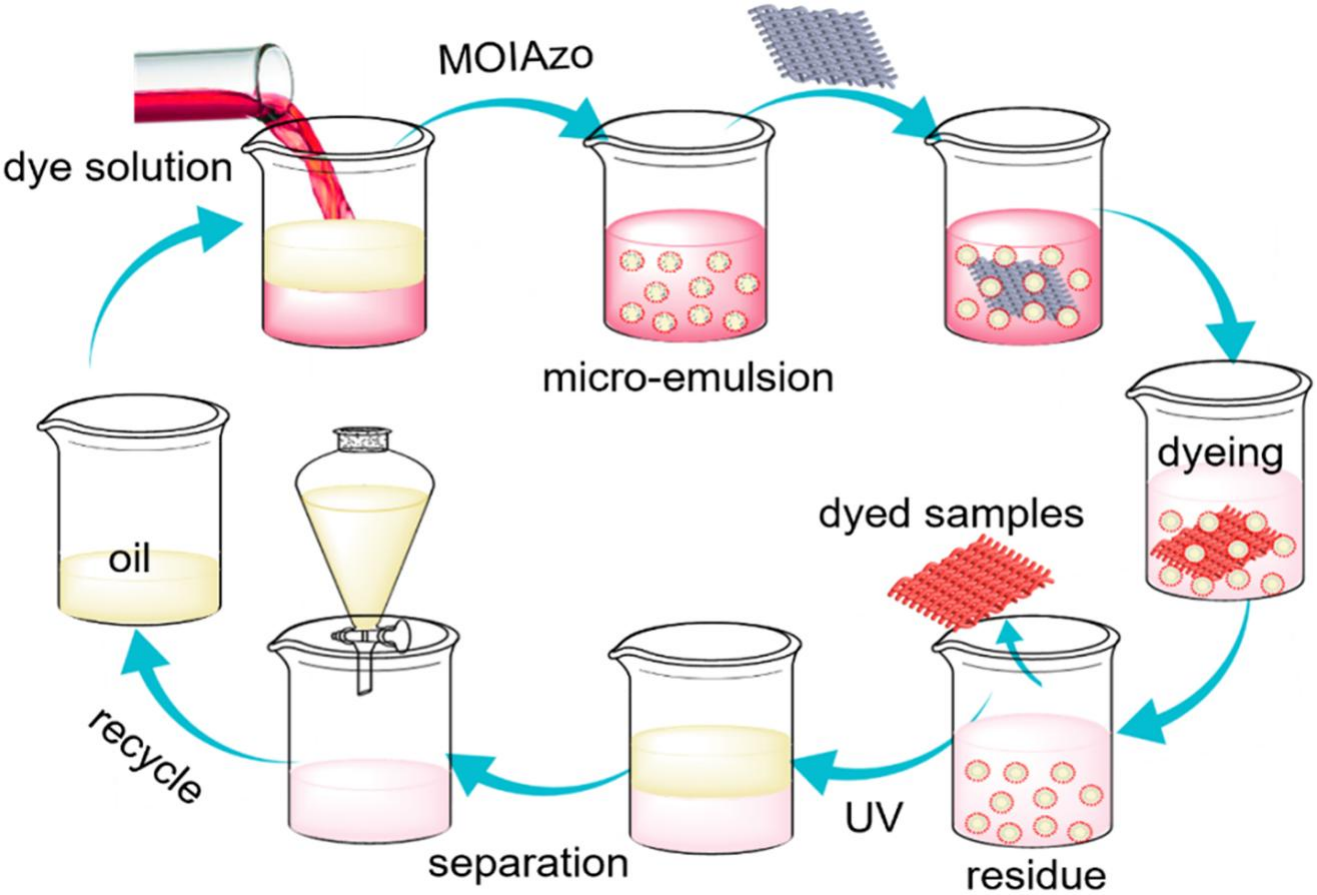
Overview of the water‐saving dyeing process utilizing emulsions.

As an example of applications, the low‐water dyeing process of cationic dyes for CDP fabric was carried out in a paraffin‐in‐water smart emulsion as the dyeing medium. The paraffin‐in‐water smart emulsion was used as a mass and heat transfer medium in the dyeing process. As illustrated in Figure 6, for the emulsifying effect of MOIAzo molecules, the dye dissolved in water and formed a stable oil‐in‐water emulsion with an equal amount of oil. This emulsion replaced water as the dyeing medium. After the dyeing, the oil and water were separated by the UV irradiation. The separated oil can be reused for the next cycle of the emulsion dyeing. Compared with traditional dyeing techniques, the emulsion dyeing will save half of the water usage, and at the same time, the discharge of wastewater is also reduced by half.

**FIGURE 7:**
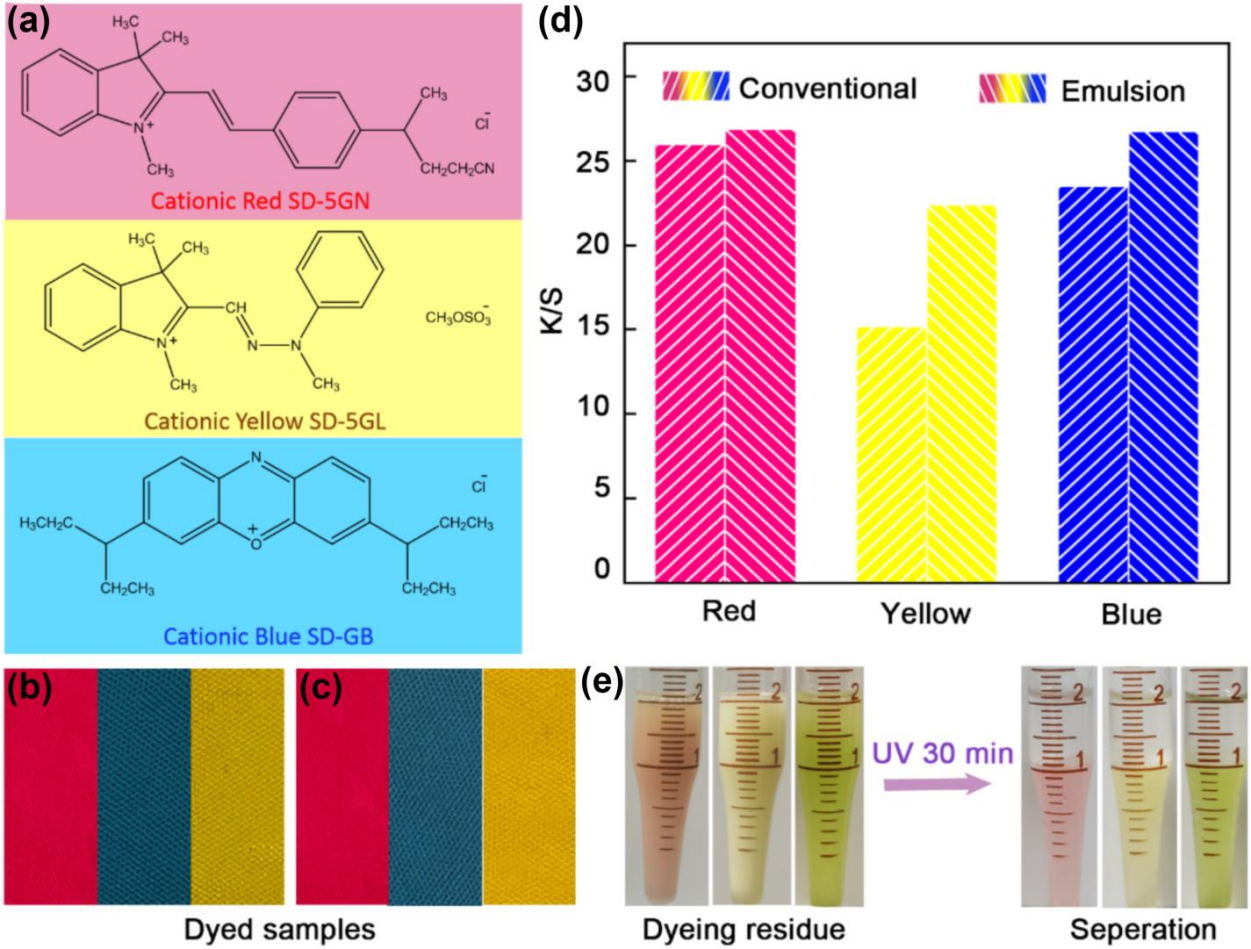
Smart emulsion dyeing method. (a) The chemical structures of the cationic dyes. (b) Photos of the dyed fabrics obtained with the conventional water bath and (c) the smart emulsion dyeing. (d) K/S values of the dyed fabrics. (e) Photos of the dyeing residue before and after the UV light irradiation.

We chose cationic dyes, Bright Red SD‐5 GN, Yellow SD‐5 GL, and Blue SD‐GB. The MOIAzo molecule and dye molecules are both cationic, so they will not interact due to the repulsion between charges. This will not affect the emulsifying performance of MOIAzo, nor will it affect the dyeing performance of cationic dyes. The chemical structures of the dyes are shown in Figure 7a. Following the conventional water bath dyeing method, we replaced water with the smart paraffin‐in‐water emulsion as the dyeing medium. The dyeing results with the two mediums were compared. Photos of the dyed fabrics obtained with the conventional water bath and emulsion dyeing conditions using the three dyes are shown in Figure 7b,c, respectively, where the amount of dye used was 2%(o.w.f). To the naked eyes, the depth and uniformity of color on the fabric dyed with the emulsion are not much different from those of the fabric dyed in the conventional water bath. Furthermore, the K/S values of the dyed fabrics with two mediums are shown in Figure 7d. The K/S value refers to the Kubelka‐Munk function, which is a measure of the color strength of a dyed material. The higher K/S values indicate deeper or more intense coloration. Compared to the dyed fabrics in a conventional water bath, the K/S values of the dyed fabrics with the smart emulsion were all higher for three cationic dyes. Particularly for Yellow SD‐5 GL, the color depth of the fabric dyed with the emulsion was 47% higher than that of water bath dyeing. Therefore, although the emulsion dyeing only uses half water of the conventional water bath dyeing, the color depth is higher, which means that the utilization rate of the dye has improved.

The residual liquid after the smart emulsion dyeing was irradiated for 30 min with the UV light. The oil phase and the water phase were completely separated, as shown in the photos in Figure 7e. By this way, the oil phase can be separated out and used for the next emulsion dyeing. Therefore, using the smart emulsion as a dyeing medium greatly reduces water consumption and represents a new dyeing process in line with green sustainable development.

The smart emulsion system was effectively utilized in a water‐saving dyeing process for cationic dyeable polyester fabrics. This process requires significantly less water and inorganic salts than conventional methods, and the emulsion allows for efficient separation and reuse of the oil phase after dyeing, highlighting its environmental benefits. The design and synthesis of MOIAzo and its application in the smart emulsion system represent a noteworthy advancement in green chemistry, offering a new approach to sustainable and efficient industrial processes.

## 4 CONCLUSIONS

An ionic liquid crystal with light triggered switch based on azobenzene groups, MOIAzo, was synthesized. MOIAzo exhibited both photoinduced and thermally induced liquid crystal properties. It could undergo a reversible liquefaction and solidification upon the light irradiation at room temperature, facilitated by the trans‐cis isomerization. Remarkably, the liquefaction temperature of the ionic liquid crystals was photoinduced to reduce reversibly by 70°C. MOIAzo as a smart emulsifier stabilized the emulsion of oil in water, such as paraffin‐in‐water, toluene‐in‐water, and n‐heptane‐in‐water, and its photoinduced structural transformation enabled light‐triggered emulsification and demulsification of the emulsions. The water‐saving dyeing of cationic dyes for CDP fabric was realized by using the paraffin‐in‐water smart emulsion as the dyeing medium. The water consumption was only half of that of the conventional water bath dyeing. After dyeing, the oil and water phases of the emulsion were completely separated through the light irradiation, and the oil phase could be reused for the subsequent dyeing process. This novel emulsion dyeing method greatly reduces water consumption and wastewater discharge. MOIAzo as a novel functional ionic liquid crystal meets the development concept of green and sustainable chemistry.

## CONFLICT OF INTEREST STATEMENT

The authors declare no conflicts of interest.

## ETHICS STATEMENT

Not applicable.

## Supporting information

Supplementary Information S1

## Data Availability

Supporting Information is available from the Wiley Online Library or from the author.
