# Supplementary material for: Smart emulsion system driven by light‐triggered ionic liquid molecules and its application in eco‐friendly water‐saving dyeing
Source: Smart Mol. 2024 Mar 19;2(2):e20230030. doi: 10.1002/smo.20230030 (PMC12118309; doi:10.1002/smo.20230030)
Supplement: Supplementary file 1 — Supplementary Information S1 [file SMO2-2-e20230030-s001.pdf]

Supporting Information

Smart emulsion system driven by light-triggered ionic liqiud molecules and its application in eco-friendly water-saving dyeing

Aiqin Gao,[1] Jiahui Liang,[1] Mingxiao Jing,[1] Xiyu Song,[1,2] Aiqin Hou[1], Kongliang Xie[1*]

[1] State Key Laboratory for Modification of Chemical Fibers and Polymer Materials, College of Chemistry and Chemical Engineering, Donghua University, Shanghai 201620, P. R. China

[2] College of Textile and Apparel, Shaoxing University, Shaoxing 312000, P. R. China

Synthesis of MOIAzo

The synthesis procedure of MOIAzo was shown in Figure S1.

12.5 mL of 36% hydrochloric acid was dissolved in 100 mL of water. Then, p-toluidine (5.35 g, 50 mmol) was dissolved in the hydrochloric acid and cooled in an ice bath to 0-5 °C. Under stirring, a 30% $NaNO_2$ solution (11.7 g) was gradually added dropwise. After 1 h, the reaction was completed. A small amount of urea was added to remove excess nitrous acid, and the resulting pale yellow transparent diazonium salt was preserved in an ice bath.

In 200 mL of water, Na_2CO_3 (5.3 g, 50 mmol), $NaHCO_3$ (4.2 g, 50 mmol), and phenol (5.15 g, 55 mmol) were sequentially added. After complete dissolution, the mixture was cooled to 0-5 °C. The diazonium salt was slowly added dropwise to the coupling solution, and the pH value was controlled using a 10% Na_2CO_3 solution. After 1 h of the reaction, the pH value of the system was adjusted to 4-5 by slowly adding 10% acetic acid. The mixture was filtered and washed five times with water, resulting in a yellow filter cake. Drying the cake yielded a yellow powder, M1, with a yield of 91.46% and a melting point of 161-165 °C.

M1 (3.00 g, 14.09 mmol), along with a certain amount of K_2CO_3 and KI, was placed in a 100 mL single-neck flask. Then, 30 mL of acetone and 8-bromooctanol (5.88 g, 28.14 mmol) were added, and the temperature was raised to 56 °C. After reacting 14 h, the mixture was cooled. The resulting mixture was filtered, and the filtrate was rotary evaporated to obtain a deep red oily substance. The crude product was washed with 20 mL of n-hexane, and the filtered product was a yellow powder, M2, with a yield of 90.00% and a melting point of 79-81 °C.

M2 (3.55 g, 10.44 mmol) was placed in a 100 mL three-neck flask along with 35 mL of toluene. The temperature was raised to 60 °C with stirring, and then succinic anhydride (1.14 g, 11.4 mmol) was added. After reacting 5 h, the mixture was cooled to room temperature, and the solvent was removed by the rotary evaporation to obtain a yellow crude product. The crude product was recrystallized with methanol, resulting in a yellow powder, M3, with a yield of 61.66% and a melting point of 115-117 °C.

M3 (2.00 g, 4.52 mmol) and DCC (0.93 g, 4.52 mmol) were placed in a flask, and 20 mL of dichloromethane was added. Then, 8-bromooctanol (0.95 g, 4.52 mmol) was added, followed by a small amount of DMAP. After stirring at room temperature for 1 h, the reaction was completed, and the mixture was filtered to remove the white insoluble compounds. The remaining liquid was extracted three times with 10 mL of dichloromethane, washed once with water, and subjected to water removal using anhydrous sulfuric acid. The resulting mixture was filtered to remove sodium sulfate and then rotary evaporated to obtain a brownish-red oily substance. The recrystallization with methanol:n-hexane (10:1) yielded a yellow powder, M4, with a yield of 81.53% and a melting point of 57-58 °C. FT-IR ν (cm^{-1}): 2933, 2856, 1722, 1242, 1019, 848, 795, 688 (Figure S2); ^1H NMR (400 MHz, $CDCl_3$), δ (ppm): 7.92-7.93(d, 2H, Ar-H), 7.71(s, 2H, Ar-H), 7.39-7.42(t, 1H, Ar-H), 7.27(s, 1H, Ar-H), 7.01-7.03(d, 2H, Ar-H), 4.09-4.12(q, 4H, $-CH_2-$), 4.05-4.07(t, 2H, $-CH_2-$), 3.41-3.44(t, 2H, $-CH_2-$), 2.65 (s, 4H, $-CH_2-$), 2.47(s, 3H, $-CH_3$), 1.83-1.88(m, 4H, $-CH_2-$), 1.64-1.66 (m, 6H, $-CH_2-$), 1.28-1.45(m, 14H, $-CH_2-$). (Figure S3). ^{13}C NMR (400 MHz, $CDCl_3$), δ (ppm): 172.43, 161.64, 152.84, 146.89, 138.91, 131.14, 128.86, 124.72, 122.62, 120.20, 114.70, 68.29, 64.86, 64.82, 33.98, 32.76, 29.26, 29.17, 29.04,

28.63, 28.58, 28.54, 28.07, 25.96, 25.83, 25.78, 21.40. (Figure S4).

M4 (1.50 g, 2.37 mmol) was placed in a flask, and 15 mL of acetonitrile was added. *N*-Methylimidazole (0.20 g, 2.37 mmol) was slowly added, and heated to a specific temperature for 9 h. The solvent was then removed by the rotary evaporation, resulting in a yellow crude product. The recrystallization with 10 mL of ethyl acetate yielded a yellow powder, MOIAzo, with a yield of 84.84% and a melting point of 79-80 °C. FT-IR ν (cm^{-1}): 3147, 3058, 2933, 2856, 1725, 1241, 1022, 847, 730, 621 (Figure S5); ^{1}H NMR (400 MHz, $CDCl_3$), δ (ppm): 10.63 (s, 1H, -CH=), 7.90-7.92 (d, 2H, Ar-H), 7.69 (s, 2H, Ar-H), 7.37-7.41 (t, 1H, Ar-H), 7.23-7.25 (m, 3H, Ar-H, -CH=), 6.99-7.01 (d, 2H, Ar-H), 4.29-4.33 (t, 2H, $-CH_2-$), 4.11 (s, 1H, $-CH_3$), 4.06-4.09 (t, 4H, $-CH_2-$), 2.62(s, 4H, $-CH_2-$), 2.45(s, 3H, $-CH_3$), 1.82-1.91(m, 8H, $-CH_2-$), 1.61-1.64(m, 4H, $-CH_2-$), 1.32-1.37(m, 14H, $-CH_2-$) (Figure S6); ^{13}C NMR (400 MHz, $CDCl_3$), δ (ppm): 172.48, 172.45, 161.67, 152.78, 146.83, 138.93, 138.04, 131.17, 128.87, 124.74, 123.19, 122.61, 121.61, 120.17, 114.72, 68.32, 64.87, 64.70, 50.21, 36.86,30.23, 29.24, 29.19, 29.16, 28.87, 28.78, 28.56, 28.46, 26.12, 25.95, 25.82, 25.66, 21.40 (Figure S7). MS (m/z): $C_{37}H_{53}BrN_4O_5$, $[M-Br]^+$ calculated: 633.74, found: 633.40.

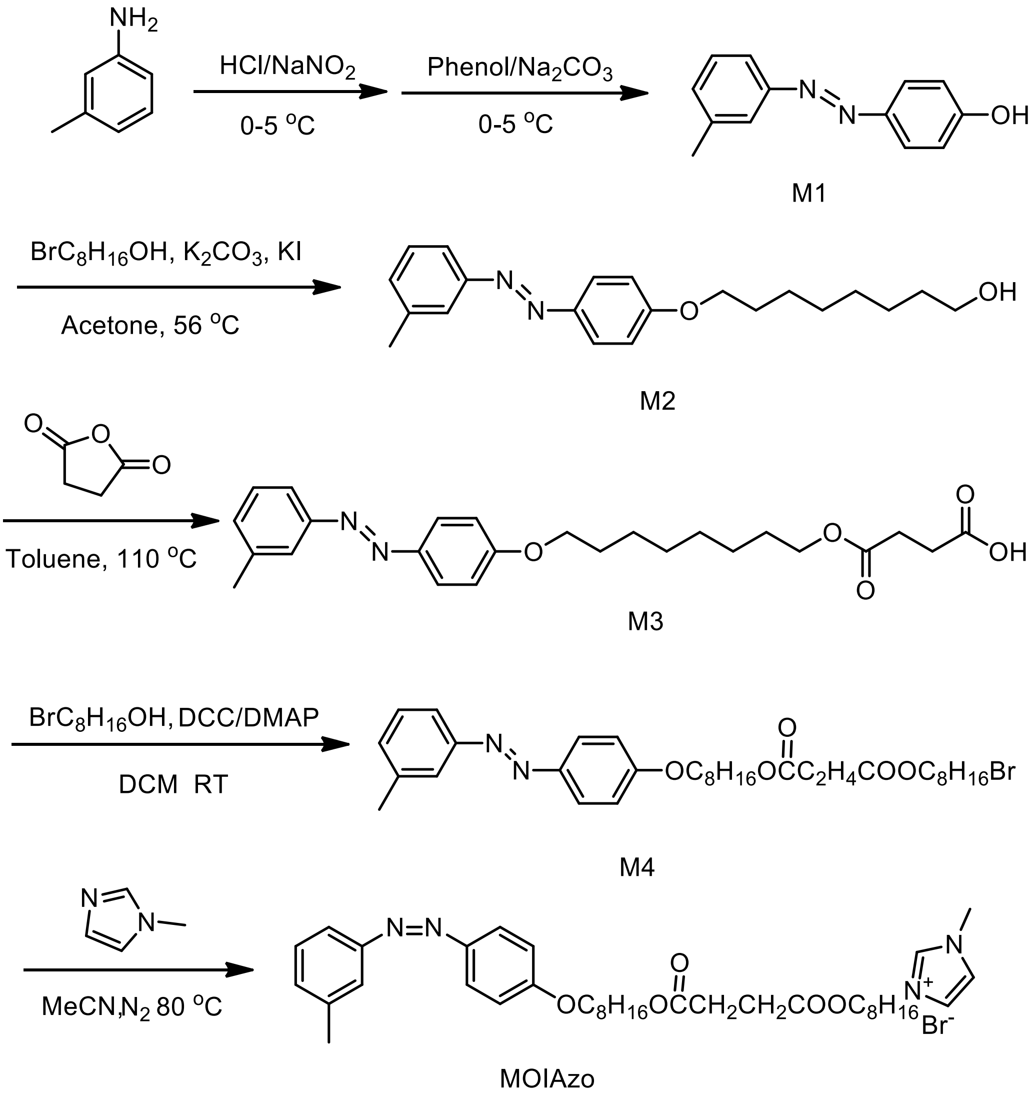

FIGURE S1 The synthesis procedure of MOIAzo.

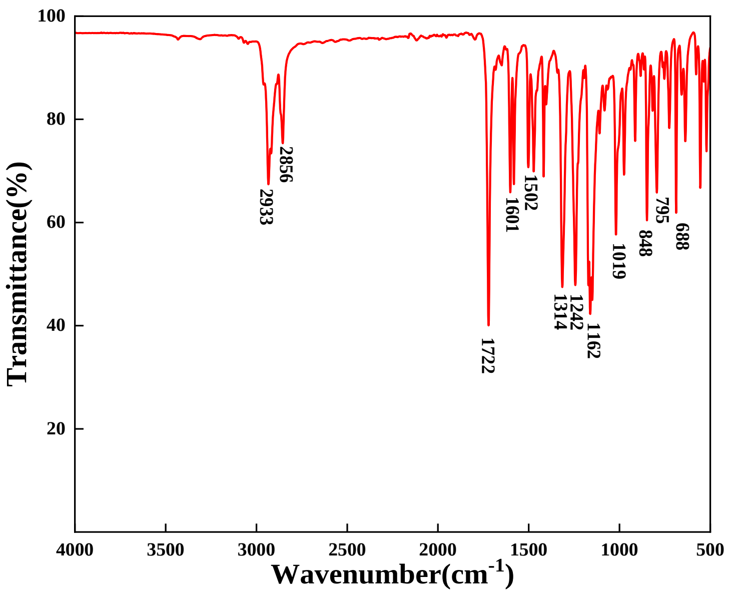

FIGURE S2 FTIR spectrum of M4.

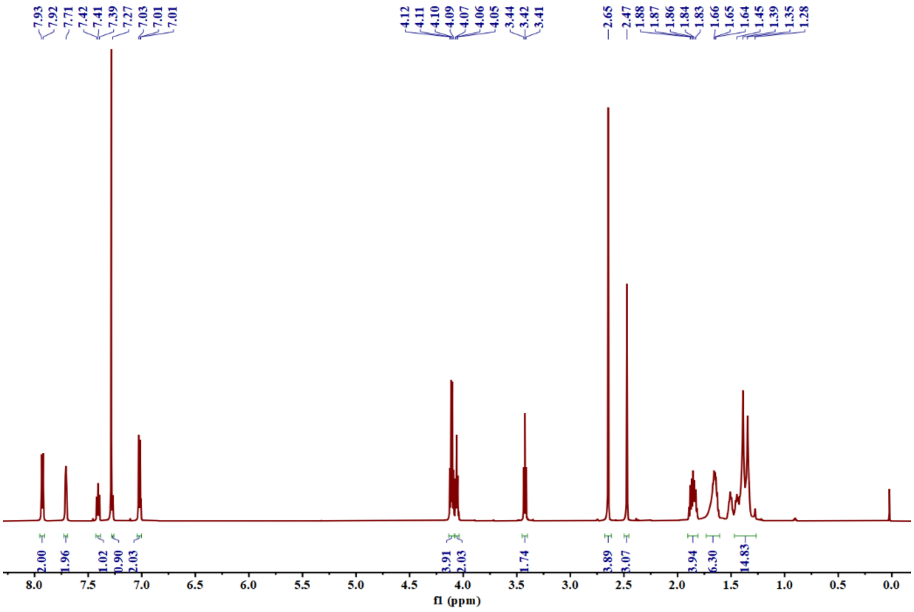

FIGURE S3 ^{1}H NMR spectrum of M4.

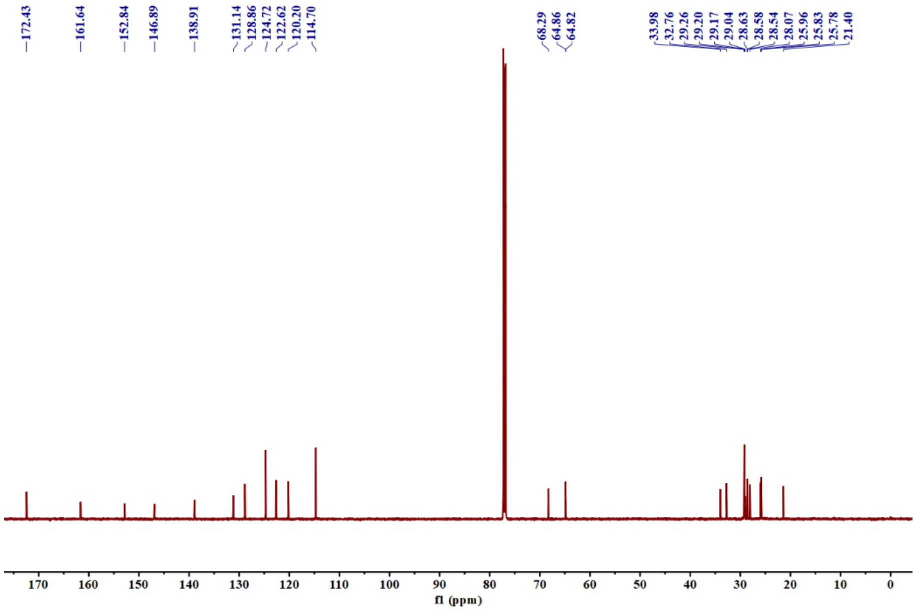

FIGURE S4 ^{13}C NMR spectrum of M4.

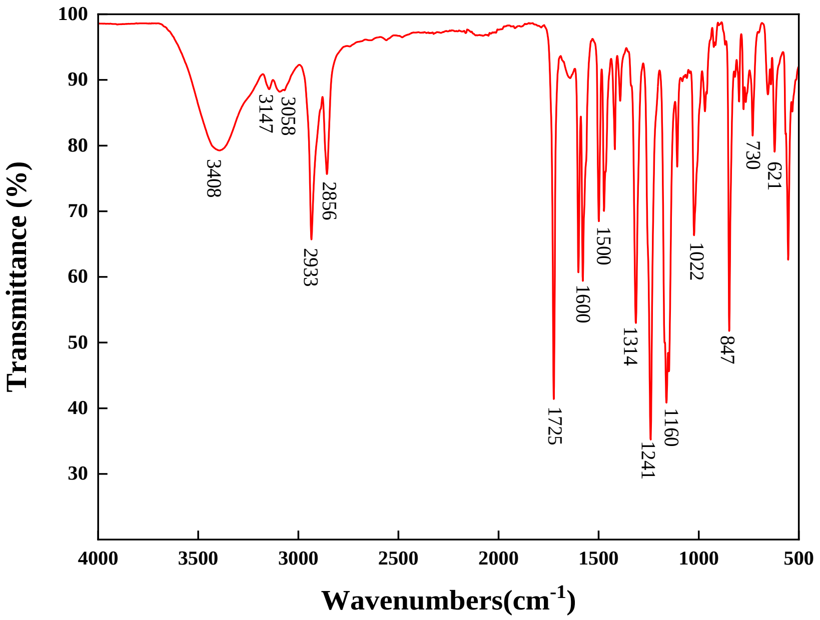

FIGURE S5 FTIR spectrum of MOIAzo.

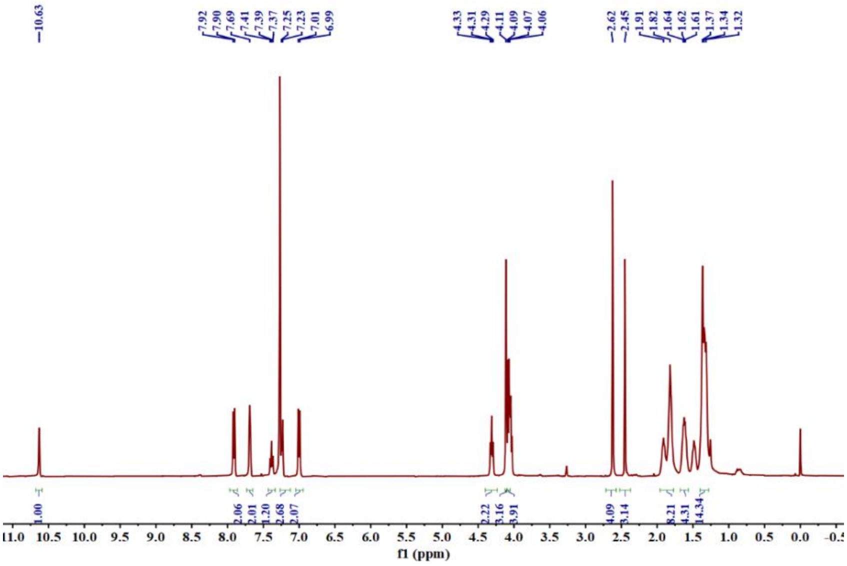

FIGURE S6 ^{1}H NMR spectrum of MOIAzo.

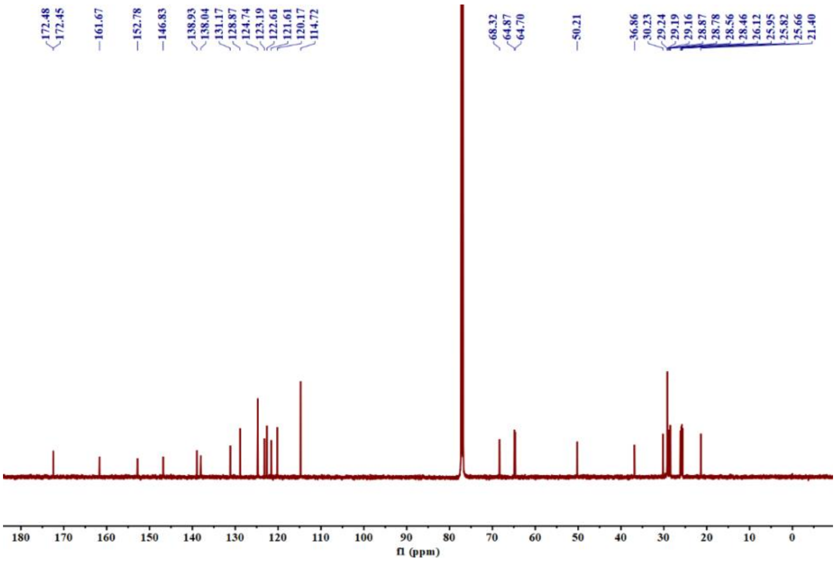

FIGURE S7 ^{13}C NMR spectrum of MOIAzo.

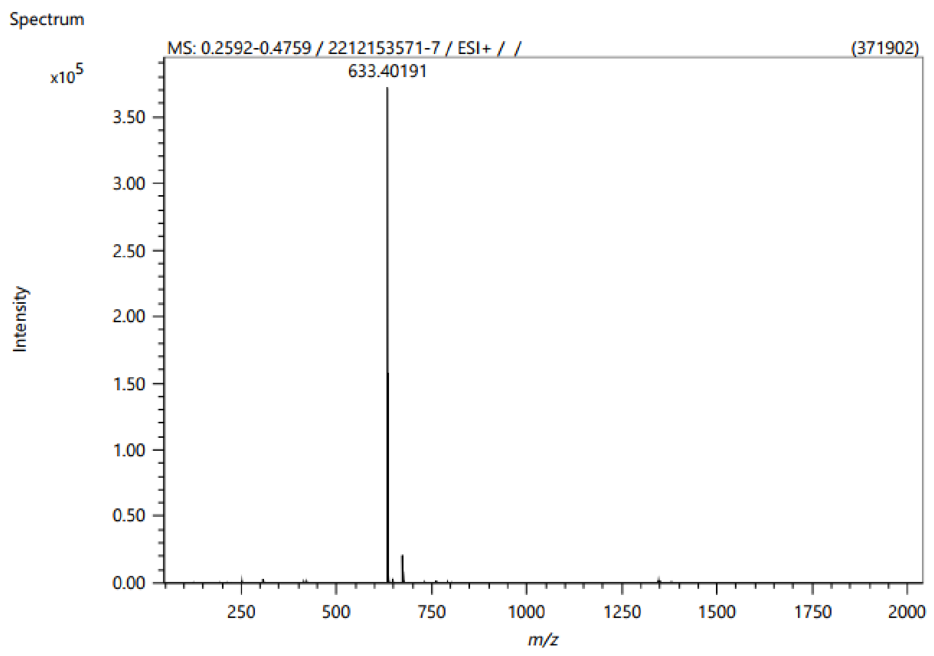

FIGURE S8 The high-resolution mass spectrometry of MOIAzo.

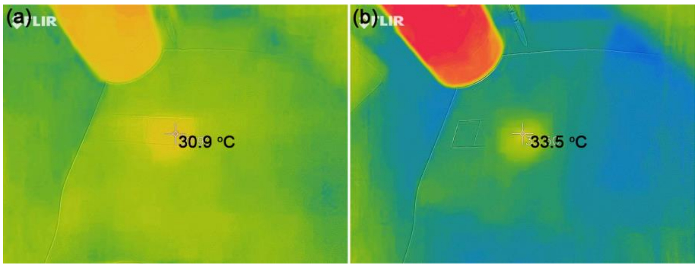

FIGURE S9 Infrared thermal images of MOIAzo (a) before and (b) after the UV irradiation for 90 min.

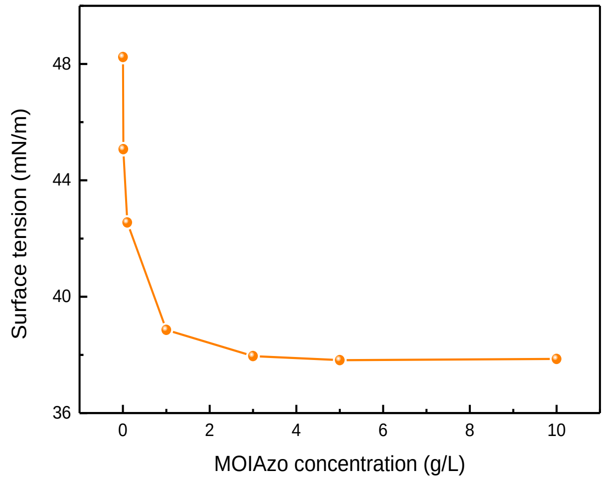

FIGURE S10 The change of the surface tension of MOIAzo aqueous solution with concentration.

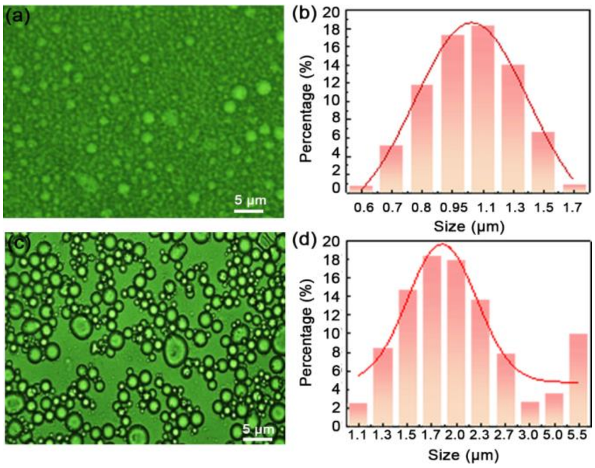

FIGURE S11 Optical microscopic photograph of (a) the toluene-water (v/v=1:1) and (c) n-heptane-water (v/v=1:1) emulsion emulsified by 0.5 g/L of MOIAzo, (b) and (d) are the corresponding particle size distribution of the emulsions.

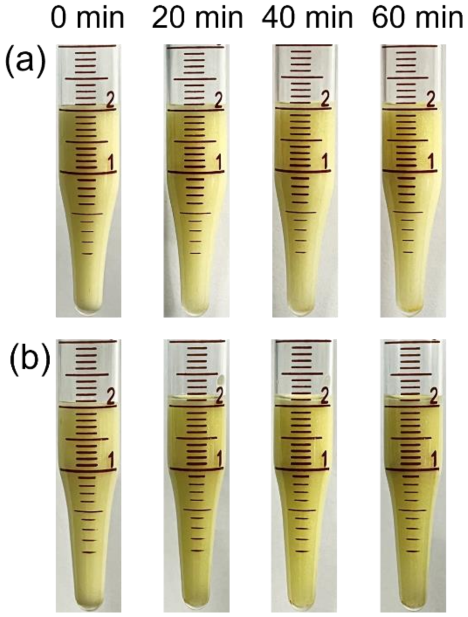

FIGURE S12 Thermal stability of paraffin-water emulsion with 0.5 g/L of MOIAzo (a) 25 ºC (b) 60 ºC.

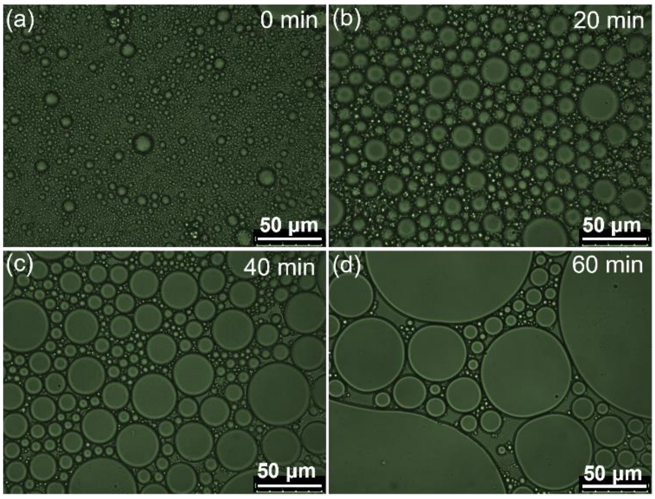

FIGURE S13 Optical microscopic images of the photo-switching process of the paraffin-water emulsion with 0.5 g/L of MOIAzo during UV light irradiation.
